# Epidemiology and clinical severity of the serotypes of human parainfluenza virus in children with acute respiratory infection

**DOI:** 10.1186/s12985-023-02214-9

**Published:** 2023-10-26

**Authors:** Le Wang, Sukun Lu, Yinghui Guo, Jianhua Liu, Peng Wu, Shuo Yang

**Affiliations:** Institute of Pediatric Research, Children’s Hospital of Hebei Province, 133 Jianhua South Street, Shijiazhuang, Hebei Province 050031 China

**Keywords:** Human parainfluenza virus, Acute respiratory infection, Serotypes, Children

## Abstract

**Background:**

Acute respiratory infections (ARI) are a threat to human health and survival, resulting in many paediatric hospitalisations. However, the epidemiological and clinical severity characteristics of the human parainfluenza virus (PIV), one of the most prevalent respiratory viruses, are not well understood in children.

**Methods:**

To identify the epidemiological features of PIV infection, in 2019, hospitalised children with ARI were screened using multiplex polymerase chain reaction (PCR) for PIV and 10 other common respiratory pathogens. Subtyping of randomly selected PIV-positive samples was performed using reverse transcription-PCR. Demographics, epidemiology, clinical manifestations, diagnosis, and outcomes were compared between PIV subtypes.

**Results:**

The annual detection rate for PIV was 14.9%, with a peak from April to September. Children under one year of age had the highest rate of PIV infection (45.5%) compared to other age groups. Of the 121 sequenced samples, 58.7%, 36.4% and 4.9% were positive for PIV-3, PIV-1 and PIV-2, respectively, and no PIV-4 was detected. Severe infections were associated with pre-existing underlying diseases and co-infections, but not with PIV serotype. After excluding cases of co-infection, we found that PIV-2 infection was associated with upper respiratory tract infections, whereas PIV-1 and PIV-3 mainly caused lower respiratory tract infections. Apart from the proportion of patients with fever, there were no significant differences among the three subtypes in terms of clinical symptoms, severity, and outcome.

**Conclusion:**

Here, PIV was the main pathogen causing ARI in hospitalised children. Appropriate attention should be paid to children with underlying diseases and co-infections to prevent the worsening of severe PIV infection.

**Supplementary Information:**

The online version contains supplementary material available at 10.1186/s12985-023-02214-9.

## Background

Human parainfluenza virus (PIV) is responsible for common acute respiratory infections (ARI) in all age groups, with its highest incidence usually occurring in young children [1]. The clinical features associated with PIV range from mild upper respiratory illnesses to severe pneumonia [2–4]. Although PIV infection is usually transient and mild, it can cause severe respiratory illnesses in young children or in those with underlying medical conditions [5, 6]. Currently, four major PIV serotypes (1–4) are known to cause respiratory diseases. Owing to the lack of epidemiological data on these serotypes, a clear understanding of the full clinical pattern of PIV is lacking. Data from clinical sources are needed to determine the burden of PIV-related disease and hospitalization and which age groups should be targeted for future vaccination efforts. PIV vaccine development is generally focused on PIV-3 because it is most associated with high numbers of hospitalisations [7]. Knowledge of the differences in the severity of clinical symptoms and outcomes caused by the different serotypes can assist in vaccine development strategies. This study aimed to explore the serotype-specific epidemiology of PIV in peadiatric ARI and to identify factors that may help establish clinical severity distinctions, which have been poorly described in the literature.

## Methods

### Data collection

We restricted admission records to those occurring in 2019. Samples in this study were collected as part of standard care from children with ARI aged 16 years and under (presenting with at least two of the following symptoms: cough, pharyngeal discomfort, nasal obstruction, coryza, sneeze, dyspnoea) at our hospital in Hebei, northern China, between January and December 2019. Demographic data, clinical features, underlying medical conditions, and disease severity were retrospectively retrieved and analysed.

### PIV detection

The samples were transported to the laboratory daily within the medium, and nucleic acids were extracted on each working day or stored at -80 °C for less than 48 h prior to testing. A multiplex polymerase chain reaction (PCR) called ResP-CE diagnostic panel (Health Gene Technologies, Ningbo, China) including 11 common respiratory pathogens, including PIV, was performed according to established protocols [8]. One of every 10 PIV-positive specimens was sampled for subtype identification. Four PIV types were tested for using TaqMan real-time PCR as previously reported [9].

### ARI diagnoses

ARI and severe ARI (SARI) cases were identified through a sentinel surveillance project for hospitalised SARI patients in China [10]. Children over 5 years of age are considered to have SARI when presenting with the following four clinical manifestations: (1) acute onset, (2) axillary body temperature ≥ 38 °C, (3) cough or sore throat, and (4) shortness of breath (respiratory rate ≥ 25 breaths/min) or dyspnoea. Children under 5 years of age are considered to have SARI if they have three of the following clinical signs: (1) acute onset, (2) cough or dyspnoea, and (3) one of the following signs or symptoms: (i) shortness of breath: respiratory rate > 60 breaths/min (infants < 2 months); respiratory rate > 50 breaths/min (infants 2–11 months), respiratory rate > 40 breaths/min (1–5 years), (ii) refusal to eat or they choke on milk, (iii) severe vomiting, (iv) convulsions, (v) drowsiness or coma, and (vi) chest wall depression or wheezing when calm. The underlying diseases included congenital heart disease, bronchopulmonary dysplasia, genetic metabolic disorders, neurological and muscular disorders, immunodeficiency disorders, anaemia, recurrent infections or history of previous hospitalisation, severe allergy or asthma, and a history of premature birth.

### Ethics

This retrospective study received ethical approval from the Institutional Review Board of the Ethics Committee of the Children’s Hospital of Hebei (CHH). Data access was also provided by CHH. The committee waived the requirement for informed consent because the study was retrospective, there was no risk of harm to the subjects, and all patients were anonymous.

### Statistical analysis

The χ2 or Fisher’s exact test was used for categorical variables. Non-parametric data were presented as median (first quartile, third quartile) and analyzed by the Kruskal-Wallis ANOVA with post hoc Dunn’s test. Univariate analysis was performed to identify differences between SARI and non-SARI patients. Logistic regression analysis was performed to select the variables associated with SARI. Random selection of samples for PIV typing and other analyses was performed by SPSS, version 25, and *p* < .05 was considered statistically significant.

## Results

### Epidemiology

**Fig. 1:**
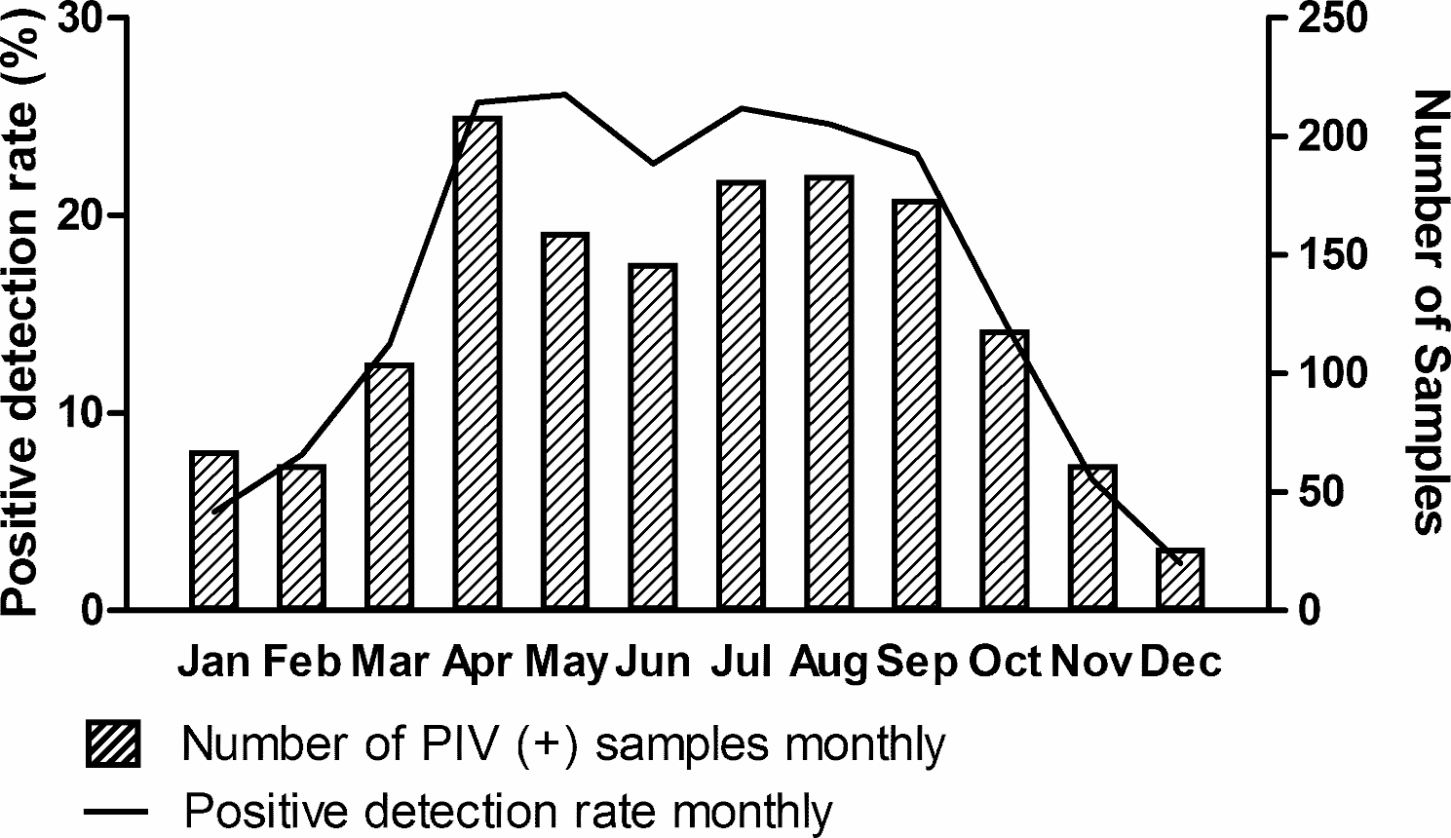
Monthly distribution of PIV in children with suspected acute respiratory infections

Among the 9,969 children admitted with suspected ARI in 2019, PIV was detected in 1,487 (14.9%) patients. A significant difference was observed between males and females (15.8% vs. 13.6%, *p* = .002). Most PIV-positive cases were found in children under 5 years of age (97.6%, 1451/1487). The median age of patients above 5 years old was 7 (quartile 6, 8) years. The positive rate of PIV among children aged < 1 year was higher than that in the other age groups (*p* < .001). The peak period for positive PIV was from April to September (Fig. 1).

### PIV Infection

Among the 1,487 PIV-infected children, 121 samples were randomly selected and tested for subtype. Of these 121 patients, 18.2% had underlying medical conditions, including congenital heart disease, asthma, developmental delay, and anaemia. The median length of stay was seven days (interquartile 5-8.5 days), and the intensive care unit admission rate was 7.5%. The majority of diagnoses were lower respiratory tract infections, with 9.1% being severe cases (11/121, Table 1).

**Table 1:** Demographic data and underlying diseases of children with human parainfluenza virus infections

		PIV infection
		n = 121
Age (year)		1.2 (0.5, 2.6)
	0–1	55 (45.5%)
	1–3	44 (36.4%)
	3–5	18 (14.9%)
	> 5	4 (3.3%)
Male gender		69 (57.0%)
Diagnosis		
Lower respiratory infection	Bronchitis	26 (21.5%)
	Pneumonia	86 (71.1%)
Upper respiratory infection	Laryngitis	6 (4.9%)
	Pharyngitis	3 (2.5%)
Severe pneumonia		11 (9.1%)
Underlying diseases		
	CHD^a^	7 (5.8%)
	Asthma	3 (2.5%)
	Development delay	1 (0.8%)
	Anemia	11 (9.1%)
Hospital duration (day)		7 (5, 8.5)

^a^ CHD congenital heart disease

Categorical data were presented as percentage (%) and analyzed by chi-square or Fisher’s test

Non-parametric data were presented as median (first quartile, third quartile) and analyzed by the Mann-Whitney U Test

Of the 11 SARI cases, 10 were children under 3 years of age and eight patients were boys. Seven were confirmed to be co-infected with other pathogens, including two cases of *Mycoplasma pneumoniae* and five cases of viruses (one with adenovirus, one with influenza A and B, two with coronavirus, and one with bocavirus). The remaining SARI cases had underlying diseases, including congenital heart disease in two cases, recurrent respiratory infection in one case, and anaemia in one case (Table S1). The non-conditional multiple logistic regression analysis of 121 cases was performed to assess factors for the differentiation of SARI and non-SARI. The underlying disease and co-infection with other pathogens possessed significantly greater predictive values as risk factors for SARI with the odd ratio (OR) values of 8.802 and 4.693, respectively (Table 2).

**Table 2:** Multivariate logistic regression analysis on severe PIV infection

Variables		B	S.E.	Wald	OR	95%CI		*P* value
						Lower	Upper	
PIV serotypes	PIV-1			2.834	-	-	-	0.242
	PIV-2	-1.676	0.996	2.834	0.187	0.027	1.317	0.092
	PIV-3	-18.26	16,094	-	0	0	-	0.999
Gender		-0.901	0.806	1.249	0.406	0.084	1.972	0.264
Age		0.174	0.159	1.202	1.19	0.872	1.624	0.273
Fever		1.125	1.068	1.110	3.08	0.380	24.963	0.292
Febrile duration before admission		-0.055	0.099	0.305	0.947	0.781	1.149	0.580
Underlying disease		2.175	0.711	9.358	8.802	2.185	35.462	0.002
Co-infection		1.546	0.718	4.631	4.693	1.148	19.19	0.031

### PIV subtyping and co-detection

Among the 121 typed PIV cases, 58.7% were PIV-3 (71/121), 36.3% were PIV-1 (44/121), and 4.9% were PIV-2 (6/121) (Table 3). None of the patients had PIV-4 or were simultaneously infected with more than one PIV subtype.

**Table 3:** Etiologic agents of 121 children with PIV infection

Etiological agents	Total	PIV1	PIV2	PIV3
	n = 121	n = 44	n = 6	n = 71
PIV mono-detection	78	32	5	41
PIV co-detection	43	12	1	30
**Virus**	**20**	**6**	**0**	**14**
PIV + HCoV	8	3	0	5
PIV + ADV	4	3	0	1
PIV + HBoV	3	0	0	3
PIV + FluB	2	0	0	2
PIV + RSV	1	0	0	1
PIV + RSV + FluB	1	0	0	1
PIV + FluA + FluB	1	0	0	1
**Bacteria**	**17**	**5**	**1**	**11**
PIV + *M. pneumoniae*	6	1	1	4
PIV + *S. pneumoniae*	5	1	0	4
PIV + *H. influenzae*	5	3	0	2
PIV + *S. aureus*	1	0	0	1
**Bacteria and virus**	**6**	**1**	**0**	**5**
PIV + RSV + *S. aureus*	1	0	0	1
PIV + RSV + *ESBL*	1	0	0	1
PIV + HCoV + *S. aureus*	1	0	0	1
PIV + HCoV + HBoV + *S. pneumoniae*	1	0	0	1
PIV + ADV + HCoV + *H. influenzae*	1	1	0	0
PIV + HCoV + *M. pneumoniae*	1	0	0	1

HCoV Human coronavirus, ADV Adenovirus, HBoV Human bocavirus, FluA influenza A virus. FluB influenza B virus, RSV Respiratory syncytial virus, PIV Human parainfluenza virus, *M. pneumoniae Mycoplasma pneumoniae*, *S. pneumoniae Streptococcus pneumoniae*, *H. influenzae Haemophilus influenz*a, *S. aureus Staphylococcus aureus, ESBL, Extended-spectrum beta-lactamases*

Of these typed cases, 35.5% (43/121) were detected simultaneously with other respiratory pathogens, of which 16.5% (20/121) were detected along with one or more viruses, and 14.0% (17/121) with one type of bacterium. The most frequently detected pathogens were human coronaviruses, *M. pneumoniae* and *Streptococcus pneumoniae* (Table 3).

### Clinical presentations and outcome

Among the three serotypes, a higher proportion of children diagnosed with upper respiratory tract infections were infected with PIV-2 (adjusted *p* = .021, Table 4). Aside from fever and respiratory rate recorded on admission (Table S2), other clinical signs (Table S2), severity (Table 4) and outcome (Table 5) of PIV infection did not vary significantly according to the individual serotypes.

**Table 4:** Diagnosis of PIV positive children

		Total	PIV1	PIV2	PIV3	*p* value
		n = 78	n = 32	n = 5	n = 41	
Severe pneumonia		4 (5.1%)	2 (6.2%)	0	2 (4.9%)	0.737
Non-severe pneumonia		74 (94.9%)	30 (93.8%)	5 (100%)	39 (95.1%)	
Lower respiratory tract infection		70 (89.7%)	27 (84.4%) ^a,b^	3 (60.0%) ^b^	40 (97.6%) ^a^	0.021^*^
Upper respiratory tract infection		8 (10.2%)	5 (15.6%) ^a,b^	2 (40.0%) ^b^	1 (2.4%) ^a^	

^a,b^ Number with the same superscript letters indicate that there is no significant difference from each other at the 0.05 level

^*^Multiplex comparison by Bonferroni showed the adjusted *p* < .05 between the two groups of PIV2 and PIV3.

**Table 5:** Treatment courses and outcomes of children with PIV infections

		Total	PIV1	PIV2	PIV3	*p* value
		n = 78	n = 32	n = 5	n = 41	
Hospitalization days		7 (5,8)	6 (5,8)	7 (4.5, 8.5)	7 (6,9)	0.535
ICU admission		6 (7.7%)	1 (3.1%)	0	5 (12.2%)	0.223
Febrile days after admission		1 (0,2.5)	1 (0, 2)	4 (1.5, 6.5)	1 (0, 2)	0.226
Antibiotics use		57 (73.1%)	26 (81.2%)	3 (60.0%)	28 (68.3%)	0.332
Methylprednisolone use		13 (16.7%)	2 (6.2%)	1 (20.0%)	10 (24.4%)	0.090
Mechanical ventilation		2 (2.6%)	1 (3.1%)	0	1 (2.4%)	0.864

ICU: intensive care unit

## Discussion

In this study, we evaluated children with ARI infected with PIV and reported the following findings: the annual detection rate of PIV was 14.9%, prevalent in the summer or early fall, with the highest yield rate in children under 1 year of age; severe PIV infection was associated with underlying diseases and co-infection with other pathogens, but not with the PIV serotype; clinical features and outcomes were similar across PIV serotypes.

In our study, PIV was detected in 14.9% of children who presented with ARI symptoms, with PIV-3 and PIV-1 being the predominant types. Recent studies have focused on the role of PIV in children with respiratory illnesses. Han et al. described seasonal trends for individual PIV serotypes and found that PIV-3 was the most frequently identified PIV serotype among 514 PIV-infected Korean children during 2015–2019 [11]. They also observed that PIV-3 was most prevalent from April to September, while other subtypes were most prevalent from September to October. DeGoote et al. reported that PIV testing increased substantially each year during 2011–2019, peaking in 2019, with PIV-3 dominating [12]. From 2013 to 2017, Gu et al. observed 22.4% (231/1029) were positive for PIV-4 which was lower than PIV-1 (31.3%) and PIV-3 (34.6%) in Korean children with respiratory tract infection [13]. Another Korean team reported that during 2015–2021, the PIV-4 accounted for 18.3% among 514 cases of PIV infection [11]. The pathogenic results of children with community-acquired pneumonia collected in China from 2014 to 2016 showed that the overall PIV positivity rate was 9% of all pathogens, with PIV3 subtype accounting for the highest percentage at 55.7%, followed by PIV1 (17.4%), PIV2 (14.6%) and PIV4 (12.2%) [14]. Epidemiological studies, such as those that focus on season or age distribution but not on the severity of PIV infection, have been reported in various locations [9, 15–17]. Our data provide a perspective on PIV activity among children with ARI and indicate the predominant prevalence of PIV-3 and PIV-1 over one year, which could help inform public health responses to PIV infection among young children that tends to circulate in summer and early fall.

In this study, approximately 98% of PIV-positive cases were found in children under 5 years of age. This implies that the younger age group remains the most common population with PIV infection, which is consistent with previous studies in the United States [12], the United Kingdom [18] and Central and South America [19]. Villaran et al. showed that 68.7% of PIV-infected children were under 5 years of age [19]. Han et al. reviewed the medical records of inpatient pediatric patients diagnosed with PIV infection during 2015–2021 and found that 74.7% of them were under 36 months of age [11]. Data from U.S. covering all age groups from 2011 to 2019 showed that the majority of positive PIV results occurred in children aged ≤ 2 years, accounting for 36% [12]. In addition, we observed that the rate of PIV positivity in children < 1 year was significantly different from that in other age groups. Similar findings have been reported in other regions. Zhao et al. reported that in England and Wales, the age group most frequently affected by PIV was children < 1 year old, followed by children aged 1 to 4 years [18]. Villaran et al. showed that PIV-3 detection rate was 4 times higher in children under 5 years old than in those aged over 5 [19]. Future studies sre needed to determine which age groups should be targeted for vaccination efforts based on the burden of PIV-related disease and hospitalization.

Despite the importance of PIV as a common cause of severe respiratory disease in children, many studies on PIV infections have focused on children with pneumonia, to the exclusion of children with other forms of ARI [20]. Although our study was limited to one centre, we included a relatively full range respiratory diseases associated with PIV, such as laryngitis and pharyngitis. We found that PIV-1 and PIV-3 were more likely to cause lower respiratory infections, while PIV-2 caused upper respiratory infections. These results have also been observed in other studies. Fry et al. observed that PIV-2 infections are usually associated with childhood croup [21], whereas PIV-1 and PIV-3 infections are related with lower respiratory diseases, including bronchiolitis and pneumonia [22]. Croup is considered to be the signature clinical manifestation of PIV-1 and PIV-2 infections [23, 24]. The inclusion of upper respiratory tract infections may prevent the underestimation of clinical symptoms caused by PIV.

Early recognition of severe illness is crucial for preventing a poor prognosis. PIV infection related to severe pneumonia has been addressed in children [9, 17, 20, 25], but few studies have considered PIV serotypes as possible risk factors [1, 9, 15]. A multi-country case-control study of children with severe pneumonia illustrated that infection with PIV subtypes 1 and 3 is a risk factor for severe pneumonia [1]. Another multicenter study from China enrolled about 5,000 ARI patients (including children and adults) and reported no difference in symptoms regardless of which of the four PIV types [26], which is consistent with our report and Howard’s [20]. Howard et al. compared the clinical symptoms across pediatric patients and found that individual clinical features of PIV infection varied little among the four serotypes [20]. The different results in these reports may arise from the different patient selection criteria and inclusion of co-infected patients [27]. In the present study, a multiplex-PCR platform was used to simultaneously test 10 types of other common respiratory pathogens, allowing an opportunity to differentiate between the impact of a single PIV infection or co-infection.

Furthermore, we found that co-infection with other pathogens is a risk factor for severe illness. Although the data are limited, Derek et al. revealed an association between bacterial co-infection with severe and necrotising pneumonia associated with PIV [28]. PIV-1 infection can cause secondary bacterial pneumonia in the elderly [29], but this association has not been well described in children. Drews et al. reviewed eight epidemiological studies and found that patients with double respiratory viral infections were hospitalised significantly more often than those with PIV infection alone [30]. Several studies focusing on influenza viruses have documented worse outcomes in children with co-infections than in those without co-infection. In a review summarizing the results of in vitro and animal studies, it was suggested that when bacteria and influenza viruses are co-infected, their virulence is enhanced and they have a synergistic effect on the host organism [31]. Together with our findings, antibiotic agents against potential bacterial co-infections should be initiated to prevent the development of severe PIV infections.

The limitations of this study include retrospectively collected data, single-centre study, and small sample size with subtype data. In addition, our observation period covers 2019 and the impact of SARS-CoV-2 circulation on PIV should be noted. Due to the pandemic of COVID-19, only 3 and 26 episodes were identified in 2020 and 2021, respectively [11]. From 2015 to 2019, the rate of PIV positivity in the United States increases substantially each year, peaking at 2019 [12] and declining in early 2020 before rising in the spring of 2021 [32]. These results suggest that SARS-CoV-2 may have already had an impact on the PIV epidemic by the end of 2019. Furthermore, all children included in this study were newly diagnosed with ARI, with a short observation period and limited available diagnostic methods, making it difficult to distinguish whether the primary cause of infection was PIV or a co-infected pathogen.

## Conclusions

In conclusion, PIV is prevalent mainly in the summer and autumn, with young children being the main affected group. The incidence of severe disease is high in children with underlying diseases and co-infections. Serotypes of PIV cause similar clinical features and outcome in hospitalised children with ARI.

### Electronic supplementary material

Below is the link to the electronic supplementary material.

Supplementary Material 1

Supplementary Material 2

## Data Availability

The datasets used and/or analysed during the current study are available from the corresponding author on reasonable request.

## List of abbreviations

ARI: Acute respiratory infection
CHH: Children’s Hospital of Hebei
PCR: Polymerase chain reaction
PIV: Human parainfluenza virus
SARI: Severe acute respiratory infection
HCoV: Human coronavirus
ADV: Adenovirus
HBoV: Human bocavirus
FluA: Influenza A virus
FluB: Influenza B virus
RSV: Respiratory syncytial virus
*M. pneumoniae*: *Mycoplasma pneumoniae*
*S. pneumoniae*: *Streptococcus pneumoniae*
*H. influenzae*: *Haemophilus influenza*
*S. aureus*: *Staphylococcus aureus*
*ESBL*: *Extended-spectrum beta-lactamases*
