# Supplementary material for: Epidemiology and clinical severity of the serotypes of human parainfluenza virus in children with acute respiratory infection
Source: Virol J. 2023 Oct 26;20:245. doi: 10.1186/s12985-023-02214-9 (PMC10604402; doi:10.1186/s12985-023-02214-9)
Supplement: Supplementary file 1 — Supplementary Material 1 [file 12985_2023_2214_MOESM1_ESM.docx]

Table S1. Clinical features of severe PIV infection cases.

| Case | Gender | Age (Month) | Sepsis | PIV subtype | Co-infection | Underlying Disease | Radiology | Complication | ICU | CPAP | Hospital Days | Outcome |
| --- | --- | --- | --- | --- | --- | --- | --- | --- | --- | --- | --- | --- |
| 1 | M | 153 | N | 3 | MP | N | Pleural effusion | N | N | N | 23 | Healed |
| 2 | F | 6 | N | 3 | MP | Anemia | Respiratory failure | Myocardial injury | Y | Y | 38 | Healed |
| 3 | M | 21 | Y | 3 | InfA+InfB | N | Multiple lobar infiltrate | N | N | N | 14 | Healed |
| 4 | M | 6 | N | 3 | HCoV | Anemia | Dyspnea | Myocardial injury | N | Y | 7 | Healed |
| 5 | M | 4 | N | 3 | HCoV | CHD | Multiple lobar infiltrate | N | N | N | 21 | 30-day readmission |
| 6 | M | 7 | Y | 3 | HBoV | N | Multiple lobar infiltrate | N | N | N | 7 | Healed |
| 7 | F | 35 | Y | 3 | ADV | N | Bronchitis | Otitis media | N | N | 9 | Healed |
| 8 | M | 3 | N | 3 | N | CHD | Multiple lobar infiltrate | N | N | N | 10 | Healed |
| 9 | M | 17 | N | 3 | N | Anemia | Pleural effusion | Otitis media | Y | Y | 29 | Healed |
| 10 | M | 9 | N | 1 | N | CHD | Multiple lobar infiltrate | Myocardial injury | N | N | 14 | Healed |
| 11 | F | 4 | N | 1 | N | Recurrent ARI | Multiple lobar infiltrate | N | N | Y | 5 | Healed |
