# Supplementary material for: Epidemiology and clinical severity of the serotypes of human parainfluenza virus in children with acute respiratory infection
Source: Virol J. 2023 Oct 26;20:245. doi: 10.1186/s12985-023-02214-9 (PMC10604402; doi:10.1186/s12985-023-02214-9)
Supplement: Supplementary file 2 — Supplementary Material 2 [file 12985_2023_2214_MOESM2_ESM.docx]

Table S2. Clinical presentations of children with PIV infections.

|  |  | Total | PIV1 | PIV2 | PIV3 |
| --- | --- | --- | --- | --- | --- |
|  |  | n=78 | n=32 | n=5 | n=41 |
| **Symptom** | |  |  |  |  |
|  | **Fever ^a^** | 54 (69.2%) | 27 (84.4%) | 4 (80.0%) | 22 (53.6%) |
|  | Body temperature (℃) | 38.2±1.3 | 38.6±1.1 | 38.0±1.3 | 37.9±1.3 |
|  | **Respiratory rate per min ^b^** | 36±8 | 34±7 | 29±4 | 38±8 |
|  | Pulse per min | 135±17 | 133±16 | 123±14 | 137±17 |
|  | Sore throat | 76 (97.4%) | 32 (100%) | 5 (100%) | 38 (92.7%) |
|  | Cough | 74 (94.9%) | 29 (90.6%) | 4 (80.0%) | 40 (97.6%) |
|  | Wheezing | 14 (17.9%) | 4 (12.5%) | 0 | 9 (21.9%) |
|  | Dyspnea | 7 (9.0%) | 4 (12.5%) | 0 | 3 (7.3%) |
|  | Vomiting | 3 (3.8%) | 2 (6.2%) | 0 | 1 (2.4%) |
|  | Lack of appetite | 3 (3.8%) | 0 | 0 | 3 (7.3%) |
|  | Enlarged tonsil | 4 (5.1%) | 3 (9.4%) | 0 | 1 (2.4%) |
|  | Diarrhea | 3 (3.8%) | 0 | 0 | 3 (7.3%) |
|  | Abdominal pain | 1 (1.3%) | 1 (3.1%) | 0 | 0 |
| **Chest Radiography** | |  |  |  |  |
|  | Single lobar infiltrate | 8 (10.2%) | 2 (6.2%) | 0 | 6 (14.2%) |
|  | Multiple lobar infiltrate (Unilateral) | 3 (3.8%) | 1 (3.1%) | 0 | 2 (4.8%) |
|  | Multiple lobar infiltrate (Bilateral) | 20 (25.6%) | 11 (34.4%) | 1 (20.0%) | 8 (19.0%) |
|  | Hilar lymphadenopathy | 1 (1.3%) | 0 | 0 | 1 (2.4%) |
|  | Consolidation | 1 (1.3%) | 1 (3.1%) | 0 | 0 |
|  | Pleural effusion | 1 (1.3%) | 0 | 0 | 1 (2.4%) |

^a^ *p*=.014 between PIV1 and PIV3.

^b^ adjusted *p* value =0.024 between PIV2 and PIV3; adjusted *p* value =0.037 between PIV1 and PIV3.

The significance in other items are not observed.
